# Bidirectional Role of Pericytes in Ischemic Stroke

**DOI:** 10.3390/brainsci15060605

**Published:** 2025-06-04

**Authors:** Jingya Xu, Weiming Zhao

**Affiliations:** Department of Biochemistry, Heilongjiang University of Chinese Medicine, Harbin 150040, China; xjy9434@163.com

**Keywords:** pericyte, ischemic stroke, cerebrovascular disease, neurovascular unit, inflammation

## Abstract

Ischemic stroke (IS) is a disease characterized by disruption of blood flow to the brain, resulting in damage to brain tissue and neurological deficits. The high incidence of IS and the complexity of the underlying pathophysiology of IS have led to the need for further therapeutic development. It has been found that pericytes are indispensable multifunctional cells, which can coordinate multiple biological processes, and play a vital role in the development of IS. The purpose of this review is to provide a detailed overview of the role of pericytes in regulating vascular blood flow, maintaining BBB, regulating immune response, and promoting intracerebral fibrosis during the pathophysiological process after IS, which have dual effects on intracerebral recovery after IS. Finally, the article summarizes the current strategies targeting pericytes for the treatment of IS.

## 1. Introduction

Ischemic stroke (IS) is an irreversible brain injury caused by insufficient or interrupted blood supply to the brain [1]. The pathophysiological mechanisms underlying IS manifestation involve a highly intricate interplay, with a cascade of reactions occurring in the brain after ischemia, including Ca^2+^ overload, oxidative stress, inflammatory responses, and programmed cell death [2,3]. Despite current medical developments that allow patients with IS to receive intravenous thrombolytic therapy and endovascular therapy (EVT), half of patients still do not respond well with appropriate recanalization and prompt treatment [4]. Targeting the NVU has been considered as a new therapeutic strategy for IS because its outcomes may be affected by the components of the neurovascular unit (NVU) [5]. The NVU consists of neurons, basal lamina, endothelial cells (ECs), microglia, astrocytes, and pericytes, which are the basic structural and functional units that make up the central nervous system (CNS). Pericytes are important participants in pathophysiologic processes after IS [6].

## 2. Pericytes

Pericytes, also known as Rouget cells, are named “pericytes” because of their anatomical location in the perivascular area around small arterioles and capillaries and their proximity to ECs [7]. They are distributed throughout the vascular system and are multifunctional cells that exhibit significant heterogeneity and regulate the associated tissues accordingly [8].

Pericytes exhibit significant molecular heterogeneity both between and within tissues [9]. To meet tissue-specific demands, the distribution and density of pericytes vary significantly across organs and vascular beds. For instance, pericytes in the CNS form tight and continuous coverage around ECs, whereas those in the perisinusoidal spaces of the liver exhibit a loose and discontinuous arrangement around ECs [10]. The heterogeneous nature of pericyte populations precludes reliable identification through any singular molecular signature, given their overlapping antigenic profiles with vascular smooth muscle cells and others [11,12]. Taking a look at the reports of the cell markers, current studies have summarized some conserved markers of pericytes in different organs of human species, such as pericyte markers in the brain of human species as RGS5, HIGD1B, SLC19A1, etc. [13,14,15,16], whereas the study of specific pericytes still requires detailed intra-tissue characterization.

They are multifunctional cells essential to the microcirculatory vascular wall that exhibit specific stem cell properties and can orchestrate several biological processes, including four aspects of blood–brain barrier (BBB) composition: maintenance of CNS homeostasis, angiogenic stabilization and blood flow regulation, tissue repair and regeneration, and modulation of immune responses [17,18].

### 2.1. Pericytes Form the BBB and Maintain CNS Homeostasis

**Figure 1:**
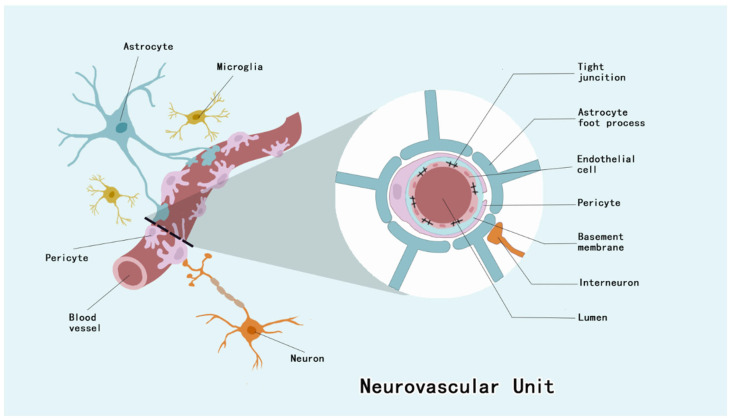
NVU is composed of ECs, neurons, astrocytes, microglia, basement membrane, and pericytes. Among them, pericytes, ECs and their tight junctions, basement membrane, and the end-feet of astrocytes form the BBB.

Pericytes of the CNS are located between the ECs and astrocyte end feet [19]. They invest approximately four–fifths of the CNS microvascular system, with regional variations observed across distinct neuroanatomical compartments, and the EC–pericyte ratio of the CNS microvascular system is 1:4 to 1:1 [19,20]. Pericytes constitute integral structural constituents of the NVU, forming continuous membrane appositions with endothelial cells that are essential for BBB integrity maintenance [21] (Figure 1). As a barrier to prevent harmful substances from entering the brain, the BBB maintains CNS homeostasis, and this barrier dysfunction has been associated with a variety of neurological disorders [22].

All organs have pericytes, but the brain has more pericytes than any other organ [23]. They have a highly heterogeneous origin, with forebrain pericytes originating predominantly from neural crest cells; pericytes in other parts of the brain are derived primarily from stem cells, in addition to studies suggesting that those cells may originate from macrophages [24]. There are three types of pericytes in the CNS: arterial pericytes, capillary pericytes, and pericytes of small veins. Pericytes surrounding small arteries express high levels of α-smooth muscle actin (α-SMA), which regulates cerebral blood flow [25]. Pericytes of capillaries are located between the capillary beds and exhibit more longitudinal protrusions that crucial for orchestrating BBB homeostasis [5]. Pericyte subtypes of small veins with stellate morphology regulate the infiltration of peripheral immune cells into the brain parenchyma [5,23,26]. Pericytes distributed in the CNS as an important cellular component of the NVU and BBB, which regulate cerebral blood flow, maintain the BBB, and contribute to neuroinflammatory processes by presenting antigens and phagocytizing substances [27,28]. They have stem cell properties, and stimulation will induce their differentiation into smooth muscle cells, fibroblasts, ECs, astrocytes, microglia, and even neurons [29,30]. Furthermore, it has been shown that decreased platelet-derived growth factor receptor β(PDGFRβ) + pericyte increases BBB permeability and is exacerbated under hyperglycemic conditions [31].

### 2.2. Pericytes and Angiogenesis and Blood Flow Regulation

**Figure 2:**
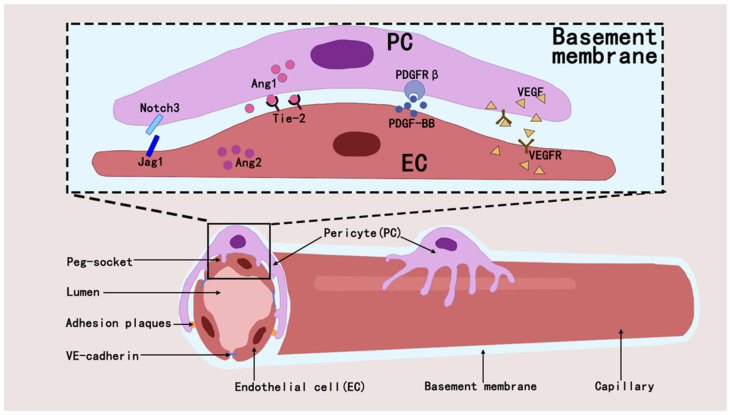
Pericytes establish interactions with ECs through diverse mechanisms, which in turn affect the modulation of intraluminal blood flow and the process of angiogenesis within their shared microenvironment.

Pericytes regulate the developmental and formative processes in the vascular system and also play a key role in maintaining vascular stability and regulating vascular permeability [32,33]. They maintain capillary network homeostasis in tissues and organs by interacting with ECs through paracrine signaling and direct physical contact [34]. They orchestrate vascular homeostasis through a paracrine signaling axis by secreting and activating molecules such as Angiopoietin1 (ANGPT1) [32,35], transforming growth factor-β(TGF-β) [36], vascular endothelial growth factor (VEGF) [37], and Notch [38,39]. The interaction between pericytes and ECs regulates angiogenesis and blood flow regulation through signaling pathways such as PDGF-B/PDGFRβ [40], ANGPT1/Tie2 [41], RhoA/ROCK [42], and eNOS/NO [43], and that these mechanisms maintain vascular stability and function [44,45] (Figure 2).

If the two types of cells are subjected to pathological conditions and act abnormally, it will lead to histopathology, such as in the periphery of the kidney where pericytes and ECs form a peritubular capillary (PTC) network, and under pathological conditions, the pericytes can be activated, detached, and transformed into myofibroblasts, which migrate from the capillaries into the mesenchyme in isolation from the ECs, affecting the stability of the blood vessels and leading to ischemia of the renal units, ultimately leading to renal fibrosis [46,47,48]. In the pathological state of the organism, pericytes and ECs are components of the tumor microenvironment (TME), and altered interactions between the two types of cells within the TME can promote tumor growth, stimulate metastasis, and angiogenesis [49]. ECs secreting TGFβ1/2 to recruit pericytes, secreting PDGF to bind to the corresponding PDGFR-β receptor on the free pericytes, and secretion of Ang-1 and Tie2 receptors by pericytes to regulate tumor angiogenesis and maturation [35,50]. Excessive pericytes coverage in TME promotes tumor growth, and the use of imatinib was monitored to down-regulate pericyte coverage and inhibit tumor development [50].

### 2.3. Pericyte Differentiation and Tissue Repair and Fibrosis

**Figure 3:**
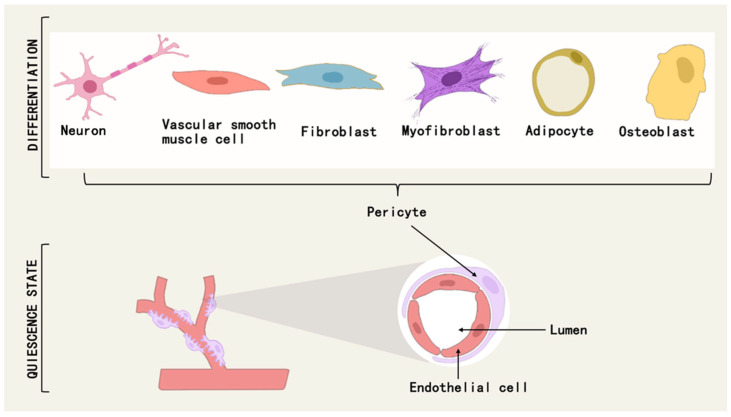
Pericytes can differentiate into osteoblasts, adipocytes, neuronal cells, myofibroblasts, etc., under different conditions.

Pericytes are a class of multipotential cells that exhibit mesenchymal stem cell (MSC)-like characteristics and therapeutic properties, and can differentiate into osteoblasts, chondrocytes, adipocytes, neuronal cells, mesenchymal stromal cells, and myofibroblasts under different conditions [51,52] (Figure 3). For example, a subpopulation of pericytes in human adipose tissue is regulated by PPARG/FABP4 interactions and is a novel source of adipocytes in the vascular microenvironment [53]. During fetal development and postnatal growth, TNAP + pericytes can differentiate into skeletal or smooth muscle cells and contribute to muscle regeneration [54], and in adulthood, myogenic pericytes can exhibit angiogenic and myogenic capacities to counteract sarcopenia due to aging [55].

This differentiation capacity of pericytes allows them to participate in the repair, remodeling, and fibrosis processes of body tissues [56]. For example, the corresponding differentiation of pericytes is involved in all stages of cardiac repair after myocardial infarction (MI), including the regulation of inflammation, fibrosis, and subsequent chronic heart failure [57]. Pericytes promote tissue healing and regeneration and have been explored for their reparative function as alternatives to stem cells [52]. During periodontitis progression, CD146/NG2 pericytes are enriched in the periodontal tissue region and can migrate to the alveolar bone surface and co-localize with ALP/OCN osteoblasts, and C-X-C motif chemokine ligand 12 (CXCL12) facilitates pericytes’ migration and increased differentiation towards osteoblasts through CXCL12-CXCR4-Rac1 signaling alveolar bone volume in periodontitis patients [58]. In bone injury, BMP-2 factor induces differentiation of pericytes to osteoblasts and accelerates bone regeneration [59,60].

They are thought to be a cellular source of pro-fibrotic tissue [61,62]. After tissue damage injury, PDGFRβ+ pericytes separate from ECs, migrate, and differentiate into α-SMA + myofibroblasts, leading to mesenchymal fibrosis [63,64,65]. The transformation of pericytes to myofibroblasts is marked by the expression of α-SMA by fibroblast-like cells, which positively correlates with the deposition of extracellular matrix (ECM) as well as the degree of fibrosis [66].

Fibrosis has a restorative role in the acute phase of injury, with deposition of collagen and others supporting the maintenance of injured organ structures, but in conditions of excessive inflammation, excessive collagen deposition and scar tissue formation are observed [67]. This pathological fibrotic process is manifested in several tissues and organs, such as the abnormal transdifferentiation of renal pericytes characterized by PDGFRβ(+)/neuroglial antigen 2 (NG2)(+) expression to myofibroblasts of the α-SMA phenotype [68], which plays a decisive role in renal mesangial fibrosis [65,69]. Hepatic stellate cells (HSCs) are activated by certain stimuli and then act as myofibroblast precursors to produce extracellular matrix pro-inflammatory/pro-fibrotic cytokines, with 82–96% of myofibroblasts shown to be derived from HSC [70,71,72]. It is also a major source of cancer-associated fibroblasts (CAFs) [73]. PDGFRβ-positive pericytes have been identified as the source of fibrotic scarring in the brain depositing fibrous ECM proteins [74]. For tissue fibrosis caused by the transformation of pericytes to fibroblasts, it has been found that drugs can be used to inhibit this process, such as imatinib, which inhibits fibrosis and inflammation caused by pericyte transformation [75]. Another example is edaravone dextroamphetamine (EDB) targeting the TGF-β1/IL-11 pathway to ameliorate fibrosis due to pericyte differentiation depositing extracellular matrix proteins caused by chronic cerebral hypoperfusion [76].

In addition, metabolic reprogramming controls the fate of pericyte differentiation and targeting aberrant pericyte metabolism effectively prevents pericyte-to-myofibroblast transformation. For instance, during pericyte–myofibroblast transformation (PMT), the regulation of adenosine monophosphate (AMP)-activated protein kinase (AMPK), a metabolic pathway involved in the metabolic transition from glycolysis to FAO, inhibits PMT [77]. Pericyte pyruvate kinase M2 (PKM2) regulates pericyte glycolysis downstream of LDHA and GLUT1 transcription as well as lactate production, and the application of PKM2 nuclear translocation inhibitors (Shikonin and TEPP-46) modulates renal fibrosis by decreasing the extent of pericyte–myofibroblast transformation [78]. Hexokinase II (HKII) expression and activity increased during PMT along with glycolysis levels, and inhibition of the PI3K-Akt-mTOR pathway by LY294002 or rapamycin resulted in decreased regulation of HKII and reduced glycolysis levels PMT was inhibited [79].

### 2.4. Pericytes and Immune Regulation

**Figure 4:**
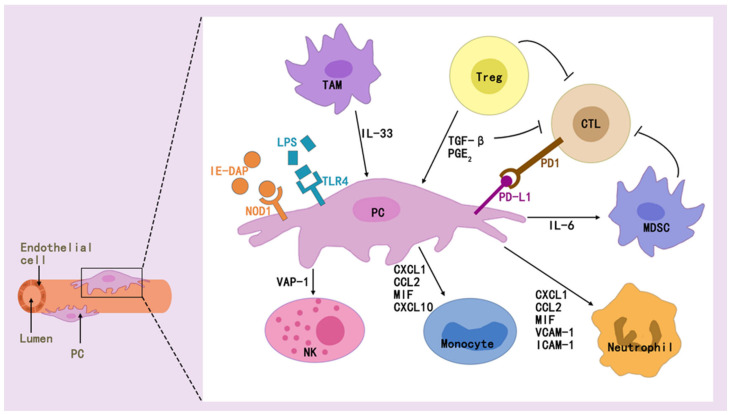
Pericytes interact with a wide range of immune cells via effectors to modulate immune effects.

Pericytes mediate immunomodulation and immunosuppression, and when stimulated accordingly, they can secrete a variety of cytokines and adhesion molecules, and these secretions play an important role in tissue-specific inflammation and immunomodulation [18] (Figure 4). Factors such as IL-6, CXC, CC, and MCP-1 are secreted by pericytes, which attract immune cells [80,81]. As sphingosine 1-phosphate (S1P) is produced in renal pericytes after kidney injury, S1P is transported to the extracellular space via spinster homolog 2 (Spns2), a pathway that enhances perivascular inflammatory signaling and promotes immune cell infiltration [82].

Pericytes can modulate innate and adaptive immunity, which is also related to the multiple effectors it secretes [83,84]. They regulate innate immunity in multiple ways, as found in exposure of pericytes to inflammatory cytokines [85], where NF-κB rapidly responds to PAMPs/DAMPs and dominates the inflammation initiation [86], STAT1 mediates macrophage polarization [87], or VCAM-1 mediates immune cell adhesion and migration [85].

It also regulate adaptive immunity and represent a new direction for immunotherapy. While pericytes preferentially regulate the induction of allogeneic regulatory T cells, unstimulated cultured tissue-derived pericytes express major histocompatibility complex (MHC) class I and inhibitory programmed cell death ligand 1/2 (PD-L1/2) molecules [88]. Immunomodulatively active pericytes generated during the intermediate phase of transplantation of the cranial neural crest can restore pulpal homeostasis during pulpitis, contributing to T-cell suppression mediated by pericytes expressing transforming growth factor β-binding protein 1 (LTBP1) [89]. Immunologic rejection after organ transplantation, long-term graft survival remains low, mainly due to T-cell-mediated acute rejection (TCMR) and antibody-mediated rejection (ABMR) [48]. Recent studies have found that early acute rejection of allografts is mediated by circulating allogeneic responsive host effector memory T cells (TEMs), and that IFN γ-activated pericytes (γ-PCs) do not inhibit cytokine production or signaling but do inhibit TEM proliferation by increasing levels of indoleamine 2,3-dioxygenase 1 (IDO1) [90]. Antibody-mediated rejection (ABMR) of the graft after organ transplantation activates pericytes within that graft to produce Notch3, whose activation promotes inflammation and fibrosis, and is associated with poor graft outcome [91].

In addition, pericytes and immune cells interact to regulate homeostasis in vivo. Pericyte-immune cell interactions can regulate glucose homeostasis, and pericytes can promote IL-1β production by immune cells to orchestrate pancreatic islet inflammation and β-cell dedifferentiation by acting with the TLR/MyD88/ Cxcl1 pathway [92]. Pericytes secrete IL-33, and type 2 innate lymphocytes (ILC2) respond to this cytokine by recruiting and activating macrophages and dendritic cells (DCs) in the pancreatic islets, and stimulation of these two immune cells in the islets indirectly promotes insulin production [93,94].

The role of pericytes in tumor immunity is also a hot topic of current research. It can promote cancer cell growth and drug resistance, as well as induce M2 macrophage polarization [95]. The presence of a population of pericyte stem cells (PeSCs) in the TME induces Ly6G cell accumulation and a decrease in the number of macrophages and dendritic cells in pancreatic cancer, and contributes to resistance to anti-PD-1 immunotherapy [96]. It was found that increasing pericyte maturation and inhibiting MEK, AKT, or Notch signaling through Rho kinase activity-related signaling pathways reversed immunosuppression and sensitized tumors to overt T-cell therapy [97]. PERICYTEs activate anti-tumor T cells through antigen presentation [98]. Glioblastoma (GB)-induced aberrant up-regulation of chaperone-mediated autophagy (CMA) contributes to the pericytes supporting the potent antitumor T-cell responses required to induce the clearance of GB [99,100]. Reduced CD8+ T-cell infiltration of renal cell carcinoma (RCC)-derived pericytes and inhibition of cell proliferation and migration of endosialin (NE)-resistant pericytes can improve the efficacy of immune checkpoint blockade therapy for RCC [101].

## 3. Pericytes and IS

### 3.1. Pericytes and Cerebrovascular Regulation and Regeneration After IS

Under physiological conditions, pericytes contract or relax according to the energy demands of the neural tissue [102]. The ischemic state can shift pericytes in diastole to systole due to the effect of constricting cerebral microvessels as a result of ROS and peroxynitrite produced during ischemia and reperfusion [102,103]. Pericyte constriction narrows capillary lumen, thereby impeding microcirculation, and abnormal pericyte constriction exacerbates capillary occlusion [103,104]. After ischemia, it contract and die rapidly, and the dead pericytes maintain the contracted state, and even if arterial flow is restored, blood flow cannot be restored to the ischemic region due to the narrowing of the lumen of the correspondingly regulated capillaries caused by pericyte death stiffness and excessive contraction, which leads to the phenomenon of no reflux after IS [105,106,107].

For the reperfusion without reflow phenomenon that tends to occur after IS, vascular regeneration allows blood to flow back to the nonperfused area through new channels [108].

Pericytes play a role in ameliorating intracerebral injury after stroke by promoting cerebral vascular regeneration, which is related to the secretion of appropriate cytokines by pericytes and their ability to have stem cell-like properties and NVU cell–cell interactions. It has been found that at this stage of IS recovery, those cells are responsible for secreting angiogenic factors, in order to promote the regeneration and stabilization of microvessels, and the stabilization of the microvascular system later promotes neurogenesis [109]. Other studies have shown that pericytes have stem cell-like properties that allow them to migrate to the site of injury, differentiate into cells needed for repair, and promote angiogenesis [110].

Specifically, neovascularization in the hypoperfused region after IS is not possible without the interaction between pericytes and ECs [111]. Neovascularization involves vessel sprouting, vessel stabilization, and vessel maturation [112]: first, under hypoxic conditions after IS, pericytes and ECs secrete matrix metalloproteinases (MMP), and the two types of cells shift to an active proliferative phenotype [113]. During this process, VEGF interacts with the fms-like tyrosine kinase 1 (FLT1) on pericytes [113], leading to delayed proliferation of pericytes around 7 days after hypoxia, which may contribute to the delayed vascular regeneration function of pericytes [114]. When two ECs form adjacent buds and fuse to recruit more pericytes, these two cellular interactions activate response proteins, which in turn activate signaling pathways associated with these proteins, controlling pericyte proliferation, migration, and recruitment, and stabilizing the neovascularization [30]. Finally, the maturation process of neovascularization is mediated by EC and pericyte secretion of factors such as angiopoietin 1 (ANG1), which mediates the further differentiation and survival of these two types of cells [115,116].

One study suggests that in the short term (within 24 h), pericytes mainly exert a vasoconstrictor function and exacerbate the degree of brain damage after IS, whereas in the long term (after one week), they behave in a way that promotes regeneration of cerebral blood vessels, and this regenerative benefit lasts up to 30 days after IS [117]. It has also been found that no changes in pericyte density and localization were observed in the 24 h after cerebral infarction, suggesting that perhaps the change occurred 24 h after reperfusion [109]. Related studies are scarce and further confirmation is needed to confirm the association between the bidirectional role of pericytes in blood flow regulation and the timing of IS (or reperfusion).

### 3.2. Bidirectional Action of Pericytes on the BBB Under Conditions of Cerebral Hypoxia

The BBB is a multicomponent system with a very complex molecular and structural composition, which is usually a tightly regulated barrier between blood and brain parenchyma and consists of ECs and their connecting tight junctions (TJs), astrocyte terminal peduncles, basal lamina, and pericytes [118]. Pericytes express a variety of TJ molecules, which are essential components of the TJ and whose expression levels play a critical role in regulating changes in the permeability of TJs [119,120,121]. They also express several barrier-associated transporter proteins, and these transporter proteins on pericytes may cooperate with transporter proteins on ECs to maintain the steady state of peripheral nerves and the BBB [121,122].

In the presence of ischemia, pericytes are stimulated to initiate a programmed death process or to be activated out of their original position, both of which may either be protective or detrimental to the maintenance of BBB integrity [123,124,125,126,127]. The protective aspect refers to the response of the pericyte to PDGF signaling after hypoxia, which detaches and migrates near areas of low perfusion while releasing trophic factors such as VEGF, which are protective factors for other cellular components of the NVU that maintain BBB integrity [123].

In contrast to the role of protecting the BBB, the response of pericytes to hypoxia can also lead to deleterious effects on the BBB. Pericytes can impair the integrity of the BBB by either specifically expressing relevant proteins upon hypoxic stimulation, causing loss of pericytes through detachment or death of the pericytes themselves, or by acting on other BBB components [124,125]. It was demonstrated that after ischemic injury, the number and coverage of pericytes decreased rapidly, and pericyte apoptosis and autophagy were detected from a mouse IS model, which may lead to pericyte loss and BBB destruction [124]. Another study found that Regulator of G-protein signaling 5(RGS5) expression in pericytes increased progressively in the hypoxic/ischemic environment of the brain during the early phase of IS and peaked at 12 h [125]. In the hypoxic environment, RGS5 regulates pericyte detachment from the vascular wall, leading to reduced vascular coverage, decreased vascular density, loss of TJs, and BBB breakdown, and this initial adaptive process of pericyte detachment during IS leads to aggravation of BBB collapse, brain edema, and neuronal cell death [125]. Pericyte detachment occurs a few hours before the first detection of BBB catabolism, and at 1 h after ischemic stroke, some pericytes undergo apoptosis, while others are activated to express NG2 and RGS5 to escape the apoptotic process and detach from their original position [126]. Within 3 h after stroke, they undergo morphological changes and show signs of detachment from the vessel wall, and detached pericytes can change their phenotype, potentially playing a role in inflammation and scarring after ischemic stroke [126]. BBB disruption can be detected at 12 h after IS, which may be due to the fact that pericytes are an integral part of the BBB, and their loss has been shown to exacerbate the breakdown of the BBB after stroke [127].

The latest studies have revealed that activation of hypoxia-inducible factor-1 (HIF-1) and its pathway produced by pericytes under hypoxic conditions plays an important role in hypoxia-induced vascular dysfunction, and that HIF-1 signaling disrupts endothelial tight junctions and contributes to the increase in the permeability of the BBB [128,129]. Activation of HIF-1 also mediates pericyte migration and induces their death, all of which exacerbate BBB damage [130]. Decreased expression of solute carrier family 22 member 8 (Slc22a8) specifically in peripheral cells of the mouse brain after IS may be a key mechanism for BBB disruption, and Slc22a8 and tight junction protein levels are correlated, and increased expression of Slc22a8 reduces BBB leakage, accompanied by Wnt/β- catenin signaling activation [131].

The pericytes surrounding the peri-endothelial BM layer are essential to ensure proper BM formation, while the astrocyte peri-BM layer separates the pericytes from the astrocyte end-feet that extend from the astrocyte body to form the outermost layer of the BBB [132]. Their loss during IS increased BBB permeability and led to increased fluid accumulation in the perivascular space in vivo, which on the one hand may be caused by the separation of microvascular pericytes from EC after IS, impairing microvascular integrity, and on the other hand, pericytes regulate the polarization of astrocyte terminal foot polarization and distribution through the regulation of aquaporin-4, α-syntrophin, and laminin α2 to regulate astrocyte end-foot polarization and distribution and abnormal polarization of astrocytes during pericytes loss [133]. It has been shown that pericytes after cerebral ischemia secrete proteases, such as MMP-9, thereby increasing BBB permeability [109].

### 3.3. Pericytes Bi-Directionally Regulate Intracerebral Immunity After IS

Pericytes bidirectionally regulate intracerebral immunity after IS, mainly through the direct immunoregulatory functions of the pericytes themselves, including producing and responding to inflammatory cytokines, presenting antigens, and exhibiting phagocytosis, as well as the pericytes as a constituent of the BBB-influencing immune factors in the brain by altering the integrity of the BBB [134].

To begin with, pericytes are capable of responding to pro-inflammatory signals and releasing anti-inflammatory cytokines/chemokines, and it can also express cell-surface proteins, including the expression of MHC class I molecules and MHC class II molecules [135,136]. Moreover, like microglia/macrophages, pericytes exhibit phagocytic activity in response to CNS injury, including the acquisition of a microglia-like phenotype by pericytes in the brain after IS [137]. The maintenance of homeostasis in the brain requires the BBB to control the selective entry of substances from the peripheral blood into the brain parenchyma [138]. Pericyte injury can lead to BBB dysfunction, disruption of tight junctions, disturbances in the TJs, and injuries, leading to influx of immune cells, neurotoxic molecules, and other molecules into the brain from the peripheral bloodstream, resulting in edema, increased neuroinflammation in a variety of brain disorders, and neuronal damage [7,139,140].

Pericytes involved in immunoregulation after IS have a protective effect. A study revealed the presence of specific pericyte subclusters in the brain at 12 and 24 h after IS, characterized by the up-regulation of genes mainly associated with immune responses, the most up-regulated genes being interleukin 6 (IL6), ADAMTS4, and IL11 [141]. Inflammatory factors expressed by all three genes act in both directions after IS [142]. Of these, the function of ADAMTS4 in inflammation is controversial [142], where it appears to attenuate inflammation after IS by increasing the number of microglia expressing arginase-1, while negatively affecting cerebrovascular integrity [143,144]. Studies have shown that PDGFRβ+ pericytes differentiate into microglia-like cells with acquired phagocytic activity after exposure to putative IS conditions and that they perform relevant functions [145,146]. IS leads to brain cell death, and excessive accumulation of dead cell debris beyond the phagocytic capacity of microglia/macrophages (MGs/MΦs) leads to accumulation of waste products in the brain and delays brain cell regeneration [146]. Pericytes in the soft meninges of ischemic areas increase phagocytosis activity of CD36+ MGs/MΦs present within the area and regulate brain repair [147]. PDGFRβ-positive pericytes increase the phagocytic activity of macrophages and promote the clearance of myelin debris from infarcted regions of the brain [148].

Pericytes exacerbate the inflammatory response in the brain after IS. One study found that pericytes in the acute phase of IS upregulate CCL2 levels, prompting inflammatory cells to recruit to that side of the stroke region, leading to brain edema [141]. They release ICAM and VCAM in IS, which promote leukocyte migration, and inflammatory factors, such as IL-3 and IL-9, leading to brain inflammation [149]. A study collected and analyzed pericyte-derived microvesicles in the plasma of IS patients and found that signaling molecules in microvesicles released by pericytes within the first 6 h after IS had immunomodulatory effects and suppressed inflammatory responses [150]. It showed a significant increase in the number of pericyte-derived microvesicles from 12 to 24 h after the IS, that its microvesicle secretome contained high levels of pro-inflammatory chemokines, and that there is a shift from an anti-inflammatory profile to a pro-angiogenic and pro-inflammatory profile, with the pro-inflammatory phase lasting up to 6 days [150]. Ischemia-induced damage-associated molecular patterns (DAMPs) can trigger the nuclear translocation of NF-кB and the up-regulation of pro-inflammatory factors in pericytes [151]. The Plexin-B1 (PLXNB1) receptor on the pericyte surface binds astrocyte-derived Semaphorin 4D (SEMA4D) after IS, making it a CD11b-positive inflammatory phenotype and promoting severe inflammation in the brain [152]. In another study, adherin-α5-deficient transgenic mice showed reduced infiltration of inflammatory cells (neutrophils, lymphocytes, and monocytes) in the brain after IS, suggesting that pericyte-derived laminin-α5 has a “pro-infiltrative” effect after ischemic injury [153].

### 3.4. Bifacial Role of Pericytes in Intracerebral Scar Formation and Fibrosis After IS

Fibrosis is a healing response to local fibroblast activation and synthesis of ECM when tissue injury occurs, but when the injury is severe or repetitive, the ECM continues to accumulate, leading to the destruction of the tissue structure [154]. The progression of IS-induced cerebral fibrosis, fibroblast activation protein (FAP)-α, is specifically expressed in areas of cerebral ischemia, while glial cells are activated to promote peri-neuroglial scar formation at the site of injury after CNS injury, and scar tissue prevents the diffusion of toxic substances in the CNS [155]. Excessive or persistent scar formation, leading to fibrosis, and the development of fibrosis after brain or spinal cord injury inhibit axonal regeneration and impede the recovery process, limiting the regeneration of the CNS in adult mammals [155,156,157].

Pericytes contribute to scar formation, which to some extent can lead to improved brain function after IS. In ischemic injury, PDGFRβ+ pericytes have been shown to trigger an increased fibrotic response in the CNS and increased fibronectin deposition in the ischemic region. PDGFRβ signaling-induced fibronectin production is essential for the repair process after IS, and proper repair within the infarct after stroke promotes the reorganization of the peri-infarct nerves [158]. Pericyte-mediated fibrotic repair processes within the infarct enhance oligodendritic formation around the infarcted area, as well as astrocyte proliferation, and restore brain function [159]. PDGFRβ-positive pericytes produce fibronectin, and intra-infarct deposition of fibronectin is significantly attenuated in pericyte-deficient PDGFRβ+/− mice, whereas the macrophage phagocytic activity for myelin debris encapsulated in fibronectin is significantly enhanced, and intra-infarct PDGFRβ-positive pericytes can orchestrate the remodeling of the critical ECM after IS, thereby creating an optimal environment to promote myelin debris clearance and peri-infarct oligodendrogliogenesis [160]. A recent study found that the expression of glutamate–aspartate transporter (GLAST)+ protein A-type pericytes contributes to ECM deposition in the brain parenchyma, and its extent determines the degree of ECM deposition and fibrosis after brain injury [61].

On the contrary, following IS, the persistent fibrosis along with macrophage infiltration existing within the ischemic core is harmful to the restoration of neurological function [161]. Myofibroblasts, which serve as the origin of pathological matrix collagen, have pericytes as their precursors [74]. The fibrotic reorganization of the ischemic part of the brain is linked to pericytes, and the creation of extensive fibrotic scar tissue in CNS lesions is considered to obstruct axonal regeneration and result in irreversible dysfunction [162,163]. Pericytes activate inflammation and fibrosis through TLR- and MyD88-dependent mechanisms, leading to tissue damage and massive deposition of extracellular matrix [164].

## 4. Targeted Pericyte Therapy for IS

Based on the dual role of pericytes in the post-IS period, current research has begun to conduct experiments and drug development in the corresponding direction, including the already-marketed pericytes-based drugs. 

In terms of pericyte regulation of CBF, the negative effect was the no-reflow phenomenon caused by excessive pericyte contraction after IS, and the positive effect was the interaction with pericytes and EC in NVU to promote cerebrovascular regeneration to restore CBF. Current studies have found that treatments such as Adenosine [165], Iptakalim [166], the PI3Kδ inhibitor CAL-101 [167], and Rapamycin [168] are all targeted at inhibiting the over-contraction of pericytes in the post-IS period. On the other hand, it has been found that the effective active ingredients of herbs can promote the formation of neovascularization after IS by enhancing the survival of pericytes after IS and stimulating the release of relevant angiogenic factors, thus improving the brain injury. For example, the herbs Salvia divinorum (SAL) and Panax ginseng (PNS) can be used for the treatment of IS, and it has been found that this may be due to the fact that their active ingredients, Salvia divinorum B (Sal B), ginsenoside R1 (R1), and ginsenoside Rg1 (Rg1) alone or in combination, act to increase the expression of the PI3K/AKT/mTOR pathway and to increase the levels of pericyte pro-angiogenic regulators, such as Ang-1, VEGF, etc. [169]. Another study found that ginsenoside F1 (GF1), a ginseng extract, can target microvascular endothelial cells and pericytes to move and thus promote neovascularization after IS [170]. Salvianolic acids (SALS), an extract of Salvia miltiorrhiza, elevated pericyte coverage and angiogenic factor-mediated angiogenesis to improve CBF after IS [171]. All of the above treatments are limited to laboratory studies and require further clarification based on the fact that pericytes may now have different effects on CBF at different time stages.

The protective effect of pericytes on the BBB is that their secretion of trophic factors after IS can protect BBB components. The detrimental effect refers to the catabolism of the BBB caused by the death of IS posterior pericytes as a component of the BBB itself and by the destruction of other components of the BBB in a direct or indirect manner. Therapeutic approaches in this direction at this stage are focused on maintaining BBB coverage of pericytes, including Edaravone [172] and Cilostazol [173], which promote pericyte proliferation and reduce MMP-9 production after IS. Sigma-1 Receptor [174], Perlecan [175], Estradiol [176,177], and Electroacupuncture Serum [178] attenuated apoptosis or migration of IS posterior pericytes. It has been found that novel sigma-1 receptor (σ-1R) agonists can inhibit the degree of autophagy and apoptosis in IS posterior pericytes and maintain BBB integrity [174]. 3K3A-activated protein C (APC) [179] and Metformin [180] can act with pericytes to maintain their coverage while also exerting direct neuroprotective effects. In addition, it has been found that atorvastatin promotes the expression of neuroglia antigen 2 (NG2) in IS posterior pericytes to mediate endothelial TJ formation [181]. The above research methods are all at the laboratory stage, while the protective role of pericyte-secreted trophic factors in the integrity of the BBB after IS has been poorly investigated, and there is a need to continue to explore in this area. For example, pericyte-derived TGF-β and PDGFR-β can ameliorate the reduction of TJ s (Claudin5 and Occludin) and attenuate neurological deficits after MCAO, and specific treatments can be further explored based on this finding [182].

Pericytes secrete anti-inflammatory factors to reduce inflammation and promote immunosurveillance in the brain after IS to remove large numbers of dead brain cells. However, pericytes also secrete pro-inflammatory factors and recruit large numbers of immune cells to the brain. A cell experiment revealed that Bellidifolin impeded pericytes death caused by oxygen–glucose deprivation (OGD) by reducing the production of nod-like receptor protein-3 (NLRP3), as well as reducing the production of inflammatory factors LDH, IL-1β, and IL-18 after the pyroptosis of pericytes [183]. There are now fewer therapeutic modalities that act on pericytes to reduce inflammation in the brain after IS, which is a promising direction for future research. In addition to developing pharmacological treatments, intervening in poor lifestyles is an important measure to mitigate the adverse effects of IS on people. Chronic alcohol consumption pre-activates the Toll-like receptor 4 (TLR4)/NF-κB pathway in pericytes, a process that can be exacerbated by post-IS NLRP3-triggering pericyte pyroptosis and exacerbating inflammation in the brain [184]. Cocaine is one of the risk factors for stroke [185], and laboratory studies have shown that cocaine induces pericytes to increase CXCL10 expression, which prompts monocytes to migrate into the brain to mediate neuroinflammation [186], and thus avoidance of cocaine abuse may reduce neuroinflammation after IS. All the therapeutic approaches and related mechanisms of action of IS targeting pericytes are summarized in Table 1.

Pericytes promote post-IS fibronectin production to promote brain repair, but at the same time, if this phase persists unchecked, it leads to fibrotic deposits that inhibit neural regeneration. No methods have been found to modulate pericyte fibrosis for IS-related treatment, probably because a certain degree of fibrosis is favorable for intracerebral repair after IS, whereas the exact degree of fibrosis is difficult to control with strict precision with drugs. For the bidirectional regulation of IS by pericytes, there are many more pericyte-related targets that can be used as future directions for mechanism research and drug development based on the current study. For instance, G protein signaling regulator 5 (RGS5) acts as a biomarker for pericytes, which are participants in the vascular remodeling process [187]. It acts as a hypoxia-responsive protein to antagonize the process of pericyte recruitment [125] while inhibiting pericyte detachment after IS [188]. One study found that statins modulate RGS5 [189]. Whereas statins are widely used as lipid-modifying drugs in the treatment of patients with IS, a retrospective analysis demonstrated that higher-intensity initial statin therapy after IS was associated with improved long-term outcomes [190]. Based on this, it is reasonable to hypothesize whether the therapeutic effect of statins on IS is not only limited to lipid lowering but also improves brain function in IS patients by modulating the RGS5 target in pericytes in the brain. A special focus can be placed on fat-soluble statins that cross the BBB, including atorvastatin, simvastatin, and others.

**Table 1:** The therapeutic approaches and related mechanisms of action of IS targeting pericytes.

Direction of Action	Treatment	Participants	Mechanism of Action
Regulation of blood flow and blood vessels	Adenosine [165]	In vitro, Mice	Activation of ATP-sensitive K^+^ channels (Gαs/cAMP/PKA pathway) in CNS pericytes results in pericyte relaxation, dilation of capillaries, and increased cerebral blood flow (CBF).
	Iptakalim (IPT) [166]	Mice, In vitro	By preventing the formation of the SUR2/EPAC1 complex, it promotes the opening of K(ATP) channels, thereby inhibiting pericyte contraction.
	PI3Kδ inhibitor CAL-101 [167]	Mice, In vitro	Inhibition of TNF α-induced TRPV2 expression in OGD/R-treated PERICYTEs results in inhibition of Ca^2+^ uptake and PERICYTE shrinkage.
	Rapamycin [168]	Mice, In vitro	Decreased pericyte contraction, increased the diameter of subpericyte capillaries and increased the number of open capillaries 30 min after recanalization, improving cerebral reperfusion after stroke.
	PDGF-D [191]	Mice, In vitro	PDGF-D is a specific ligand for PDGFRβ, (PDGFR) β controls pericyte survival, migration, and interaction with brain endothelial cells, and PDGF-D promotes stable neovascularization of injured tissue and improves cerebral perfusion by acting on pericytes.
	Salvia miltiorrhiza (SAL), Panax notoginseng (PNS) [169]	In vitro	Suppressed oxidative stress and apoptosis, and simultaneously enhanced the release of pericyte-derived pro-angiogenic regulators, which are related to the PI3K/AKT/mTOR and JNK/ERK/P38 signaling pathways.
	Ginsenoside F1 (GF1) [170]	Rats, Zebrafish, In vitro	Activation of the Insulin-like Growth Factor 1 (IGF-1)/Insulin-like Growth Factor 1 Receptor (IGF1R) axis promotes the recruitment of pericytes into newly formed vessels to stabilize and induce maturation of the vascular system.
	Salvianolic acids (SALS) [171]	Mice	Activation of the Janus kinase 2/signal transducer and activator of transcription 3 (JAK2/STAT3) signaling pathway increases pericyte coverage in the peri-infarct area.
Mitigation of BBB damage	Edaravone (Eda.B) [172]	Rats	Facilitates the multiplication of pericytes, augments the extent to which pericytes envelop ECs, and diminishes the generation of MMP-9.
	Cilostazol [173]	Rats	Avoid the dissociation of pericytes from microvessels, promotes pericyte multiplication, and restrains the synthesis of MMP-9. Enhances pericyte coverage in the brain.
	Dimethyl malonate (DMM) [192]	Mice	Surge in pericyte numbers.
	Sigma-1 Receptor [174]	Mice, In vitro	Through the combined function of PDGFRβ and integrin α5β1, the recruitment of pericytes is regulated, which helps sustain and repair the BBB post-IS.
	Perlecan [175]	Mice	Through the combined function of PDGFRβ and integrin α5β1, the recruitment of pericytes is regulated, which helps sustain and repair the BBB post-IS.
	Estradiol [176,177]	In vitro	17β-estradiol down-regulates miR-638 via ER-β to eliminate TNFα-induced pericyte migration in human cerebral vasculature.
	Electroacupuncture Serum [178]	Rats, In vitro	Prevents OGD/R-induced BBB damage in vitro by attenuating pericyte apoptosis and migration and enhancing pericyte viability.
	3K3A-activated protein C (APC) [179]	Mice	Maintains pericyte coverage and reduces ischaemia-induced pericyte contraction. Inhibits nuclear factor-κB-mediated BBB degradation of MMP9 enzyme expression, inhibits inflammatory cytokines, and exerts direct neuroprotective activity.
	Metformin [180]	Mice	Inhibition of JNK p38 MAPK signaling activation in ischaemia/reperfusion injury prevents pericyte apoptosis and promotes nerve regeneration.
	Atorvastatin [181]	Rats	Promotes pericyte-mediated endothelial TJ formation, which plays an important role in remodeling the vascular system.
immunomodulation	Bellidifolin [183]	In vitro	Suppresses the assembly of pro-inflammatory NLRP3/ASC/Caspase-1 complexes, thus safeguarding pericytes from undergoing cell death.

## 5. Summary and Outlook

**Figure 5:**
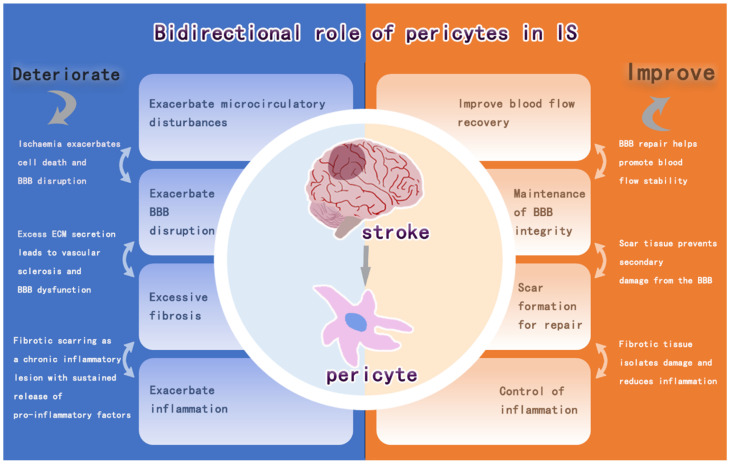
Bidirectional regulation of pericytes after IS.

Pericytes are a key component of NVU and have effects such as stem cell properties, blood flow vasoregulatory functions, and immune effects. Pericytes are involved in the mechanisms of CBF-regulated angiogenesis, BBB damage and repair, intracerebral inflammation, and pro-fibrosis in infarcted regions after IS, and these effects are manifested by the bidirectional regulation of pericytes on intracerebral repair after IS (Figure 5). The detachment of pericytes and the recruitment of inflammatory factors after IS aggravate the BBB damage and intracerebral inflammatory response, and the further fibrosis impedes the regeneration of neuroaxonal synapses in the brain. It can also promote vascular regeneration, release neurotrophic factors to stabilize the BBB and promote neurogenesis after IS. Therefore, pericytes regulate the response in the brain and have a dual role in brain damage after IS. Among these, pericytes are important for CBF, immunomodulation, and fibrosis bi-directional modulation after cerebral infarction, all of which have been found to possibly correlate with the timing of IS (or reperfusion). This includes regulation of CBF within 24 h after IS onset, which is more biased towards no reflow to exacerbate the injury and later vascular regeneration to restore cerebral blood flow [125]. Its modulation of immunity, on the other hand, is biased towards the secretion of anti-inflammatory factors at 24 h, after which it becomes a pro-inflammatory effect [158]. The time point at which the degree of fibrosis is balanced is even more difficult to control. The evidence for these findings is incomplete, and more experiments are needed to prove them.

Currently, possible therapies include acting on pericytes to improve the vascular regulation of blood flow in the ischemic area after IS and maintaining the integrity of the BBB, which have achieved certain therapeutic effects. However, there are still directions to be explored in targeting pericytes for IS treatment, including whether pericytes in NVU after IS have complex interactions with other cells, whether the bidirectional effect of pericytes on IS is related to the time of IS occurrence, and whether different drugs can be applied at different times to achieve better therapeutic effects, which need to be explored by further clinical studies in order to formulate a more accurate and effective treatment strategy. These questions need to be explored in further clinical studies in order to develop more precise and effective treatment strategies.

## Abbreviations

IS	Ischemic stroke
BBB	blood–brain barrier
EVT	endovascular therapy
NVU	neurovascular unit
ECs	endothelial cells
CNS	central nervous system
α-SMA	α-smooth muscle actin
PDGFRβ	platelet-derived growth factor receptor β
ANGPT1	Angiopoietin1
TGF-β	transforming growth factor-β
PTC	peritubular capillary
TME	tumor microenvironment TME
MSC	mesenchymal stem cell
CXCL12	C-X-C motif chemokine ligand 12
ECM	extracellular matrix
HSC	Hepatic stellate cells
CAF	cancer-associated fibroblasts
PMT	pericyte–myofibroblast transformation
AMPK	adenosine monophosphate AMP -activated protein kinase
PKM2	Pericyte pyruvate kinase M2
HKII	Hexokinase II
S1P	sphingosine 1-phosphate
Spns2	spinster homolog 2
MHC	major histocompatibility complex
PD-L1/2	programmed cell death ligand 1/2
LTBP1	transforming growth factor β-binding protein 1
TCMR	T-cell-mediated acute rejection
ABMR	antibody-mediated rejection
γ-PCs IFN	γ-activated pericyte
IDO1	indoleamine 2,3-dioxygenase 1
ABMR	Antibody-mediated rejection
ILC2	type 2 innate lymphocytes
DCs	dendritic cells
GB	Glioblastoma
CMA	chaperone-mediated autophagy
RCC	renal cell carcinoma
MMP	metalloproteinases
FLT1	fms-like tyrosine kinase 1
TJs	tight junctions
HIF-1	hypoxia-inducible factor-1
Slc22a8	solute carrier family 22 member 8
IL6	interleukin 6
MGs/MΦs	microglia/macrophages
SEMA4D	Semaphorin 4D
DAMPs	damage-associated molecular patterns
GLAST	glutamate–aspartate transporter
